# Chronic Ethanol Exposure during Adolescence in Rats Induces Motor Impairments and Cerebral Cortex Damage Associated with Oxidative Stress

**DOI:** 10.1371/journal.pone.0101074

**Published:** 2014-06-26

**Authors:** Francisco Bruno Teixeira, Luana Nazaré da Silva Santana, Fernando Romualdo Bezerra, Sabrina De Carvalho, Enéas Andrade Fontes-Júnior, Rui Daniel Prediger, Maria Elena Crespo-López, Cristiane Socorro Ferraz Maia, Rafael Rodrigues Lima

**Affiliations:** 1 Laboratory of Functional and Structural Biology, Institute of Biological Sciences, Federal University of Pará, Belém-Pará, Brazil; 2 Laboratory Pharmacology of Inflammation and Behavior, Institute of Health Sciences, Federal University of Pará, Belém-Pará, Brazil; 3 Laboratory of Molecular Pharmacology, Institute of Biological Sciences, Federal University of Pará, Belém-Pará, Brazil; 4 Department of Pharmacology, Center of Biological Sciences, Federal University of Santa Catarina, Florianópolis, Santa Catarina, Brazil; Roma Tre University, Italy

## Abstract

Binge drinking is common among adolescents, and this type of ethanol exposure may lead to long-term nervous system damage. In the current study, we evaluated motor performance and tissue alterations in the cerebral cortex of rats subjected to intermittent intoxication with ethanol from adolescence to adulthood. Adolescent male Wistar rats (35 days old) were treated with distilled water or ethanol (6.5 g/kg/day, 22.5% w/v) during 55 days by gavage to complete 90 days of age. The open field, inclined plane and the rotarod tests were used to assess the spontaneous locomotor activity and motor coordination performance in adult animals. Following completion of behavioral tests, half of animals were submitted to immunohistochemical evaluation of NeuN (marker of neuronal bodies), GFAP (a marker of astrocytes) and Iba1 (microglia marker) in the cerebral cortex while the other half of the animals were subjected to analysis of oxidative stress markers by biochemical assays. Chronic ethanol intoxication in rats from adolescence to adulthood induced significant motor deficits including impaired spontaneous locomotion, coordination and muscle strength. These behavioral impairments were accompanied by marked changes in all cellular populations evaluated as well as increased levels of nitrite and lipid peroxidation in the cerebral cortex. These findings indicate that continuous ethanol intoxication from adolescence to adulthood is able to provide neurobehavioral and neurodegenerative damage to cerebral cortex.

## Introduction

According to World Health Organization (WHO), the abusive consume of ethanol (EtOH) is responsible by over 2.5 million of premature deaths per year and almost 4% of all deaths worldwide are attributed to alcohol. Recent projections have indicated that EtOH consumption will continue to rise in coming decades, and that young adults are at highest increment in rising rates of consumption [1]. In this context, the last National Survey on Alcohol and Drug Use in Brazil [2] indicated that alcohol consumption is increasing, mainly among adolescents, becoming a serious health problem.

Some brain regions, such as the cerebral cortex, hippocampus and cerebellum, are highly vulnerable to chronic EtOH exposure and significant reduction in brain weight and volume has been found among alcoholics [3]–[5]. However, it must be stressed that brain damage severity is dependent of many variables including EtOH consumption profile (amount, frequency and duration) together with subject’s variables (age, gender, etc) [6]–[11].

It is well documented that EtOH exposure during adolescence produces neurobehavioral alterations that differ from those seen in adults and that EtOH-induced structural modifications in specific brain regions including cerebral cortex and hippocampus may underlie these cognitive and affective impairments [12]–[14]. EtOH is more deleterious to brain during adolescence probably because during this period several CNS structures are under maturation, with changes from molecular components till brain weight [15]. Although the exact molecular mechanisms associated with EtOH-induced brain damage remain to be elucidated, previous studies have demonstrated the role of neuroinflammatory and oxidative stress processes [12], [14], [16], [17].

Several studies have reported that chronic intoxication causes increased microglial activation and astrocytic hypertrophy, along with release of proinflammatory cytokines [18]–[22] and that those events may be associated with neurodegeneration induced ethanol [23].

Regarding oxidative stress, one of the main reactive nitrogen species is the nitric oxide (NO), produced by the enzyme nitric oxide synthase which is induced in pathological situations such as chronic EtOH intoxication [24].

Therefore, in the present study we investigated whether heavy chronic EtOH exposure during adolescence may induce motor impairments and histological damage in cerebral cortex of rats. We also addressed the putative involvement of neuroinflammatory and oxidative stress mechanisms in such effects of chronic EtOH exposure during adolescence.

## Materials and Methods

### Animals and experimental groups

A total of 20 adolescent male Wistar rats (35 days old), obtained from the Federal University of Pará (UFPA) were kept in collective cages (5 animals per cage). Animals were maintained in a climate-controlled room with a 12-h light/dark cycle (lights on 7∶00 AM), and food and water *ad libitum*. All procedures were approved by the Ethics Committee on Experimental Animals of the Federal University of Pará (license number BIO- 007–09) and followed the guidelines suggested by the *NIH Guide for the Care and Use of Laboratory Animals*. Animals received orally (gavage) distilled water or EtOH (6.5 g/kg/day, 22.5% w/v) (n = 10 animals) once a day, for 55 days (i.e., until the 90^th^ day of life), as previously described [14]. The current protocol of EtOH administration was based on previous studies from our group [14] showing that EtOH intoxication (6.5 g/kg/day) during the developing CNS may induce long-lasting neurobehavioral impairments and brain histochemical alterations in rats.

To investigate putative effects of EtOH intoxication on overall poor nutrition levels that may directly affect the motor performance and cortical damage, the animals’ body weight was controlled during the entire period (55 days) of EtOH exposure (i.e., from the 35^th^ day until the 90^th^ day of life) at intervals of 7 days. The animals’ survival rate in each group was assessed during the entire experimental protocol period and all animals survived the entire study. Moreover, all the behavioral and immunohistochemistry analyses were performed by an experienced experimenter who was unaware of the experimental group of the animals tested.

### Behavioral assays

Twenty-four hours after the last EtOH or distilled water administration, animals were conducted to the room assay and acclimated by at least 1 h before the beginning of the behavioral tests, with attenuation of noise levels and low illumination (12 lux).

#### Open field

The spontaneous locomotor activity of the animals was evaluated in an open field arena during 5 min. The apparatus, made of wood covered with impermeable Formica, had a white floor of 100×100 cm (divided by black lines into 25 squares of 20×20 cm) and white walls, 40 cm high. Each rat was placed at the center of the open field and the numbers of squares crossed were registered.

#### Inclined plane

The animal’s ability to maintain postural stability was evaluated with the inclined plane test. The rats were placed on the inclined plane which is raised in 5 degrees increments and can be used as an index of hind limb strength [14], [25]. The maximum inclination at which the rat could maintain its position for 5 s was recorded as the final angle. Each rat was tested in five consecutive trials with an inter-trial interval of 60 s, and the average angle was calculated.

#### Rotarod

The rotarod apparatus (Insight Scientific Equipments, SP, Brazil) consists of a grooved metal roller (8 cm in diameter) and separated 9-cm wide compartments elevated 16 cm. As a part of the test procedure, animals were initially trained to maintain themselves on the rotating rod at 15 rotation per minute (RPM) for 3 min (habituation phase). After a period of 24 h, the animals were evaluated for their ability to remain on the rotating rod during 4 successive trials of 3 min at 15 RPM with an inter-trial interval of 60 s [14].

### Histological evaluation

#### Perfusion and immunohistochemistry

Following behavioral assays, a set of animals (n = 5 animals per group) were deeply anesthetized with ketamine hydrochloride (90 mg/kg, i.p.) and xylazine hydrochloride (10 mg/kg, i.p.) and transcardially perfused with heparinized 0.9% saline solution followed by 4% paraformaldehyde in 0.2 M phosphate buffer. Surgical manipulation was performed only after both the corneal and the paw withdraw reflexes were abolished. Brains were removed from the skull and post-fixed for 12 h in the same fixative and cryoprotected in increasing concentrations gradients of sucrose-glycerol solutions over 7 days. The brain was then frozen in TissueTek, 20 µm coronal sections of cerebral cortex were cut using a cryostat (CarlZeissMicron, Germany). Sections were prepared on to gelatinized slides air dried for 24 h. They are stored in a freezer at −20°C for posterior analysis.

Sections of 20 µm were submitted to immunohistochemistry analysis as described previously [14], [26]. Briefly, the slide-mounted sections were removed from the freezer, kept in an oven at 37°C for 30 min and rinsed in 0.1 M phosphate buffer saline (PBS) for 5 min. In order to improve labeling intensity, sections were treated with 0.2 M boric acid (pH 9.0) previously heated to 65°C for 25 min. The temperature was maintained constant over the treatment period. Sections were allowed to cool down for about 20 min and incubated under constant agitation in an 1% hydrogen peroxide solution in methanol for 20 min. Sections were then rinsed in 0.05% PBS/Tween (Sigma Company, USA) solution for 5 min for three times and incubated with 10% normal horse serum (NeuN) and goat serum (GFAP and Iba1) in PBS for 1 h. Without further rinsing, sections were then incubated overnight with the primary antibody in PBS, NeuN (1∶500, Miliporere, USA), GFAP (1∶1000, Sigma, USA) and Iba1 (1∶1000, Dako, Denmark) rinsed in PBS/Tween solution for 5 min (3 times), and incubated with biotinylated horse anti-mouse (NeuN antibody) and goat anti-rabbit (GFAP and Iba1 antibodies) secondary antibodies (Vector Laboratories, USA) diluted at 1∶ 500 in PBS for 2 h. As a negative control, normal sera, rather the primary antibody, were used in some sections.

Sections were rinsed again for 5 min (three times) and incubated in the avidin-biotin-peroxidase complex (ABC Kit, Vector Laboratories, USA) for 2 h. Sections were rinsed four times (5 min each) and revealed with Diaminobenzidime (DAB) [14], [26]. After the DAB reaction, sections were rinsed two times (5 min each) in 0.1 MPB, dehydrated, and coverslipped with Entellan (Merck, Germany).

#### Qualitative and quantitative analyses

**Figure 1:**
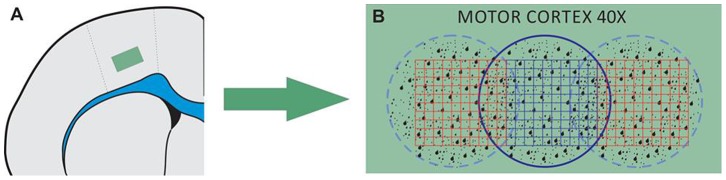
Schematic representation of the methodology utilized to count cells in the motor cortex area.

All sections were initially surveyed by light microscopy. Illustrative images from all experimental groups were obtained with a digital camera (Moticam 2500, USA) attached to a microscope (Nikon, Eclipse 50i, USA). We used coronal sections containing cerebral cortex to count the numbers of neuron (NeuN^+^ cells), microglia (Iba1+ cells) and astrocyte (GFAP^+^ cells) using a 0.0665 mm^2^ square graticule attached to the microscope eyepiece (objective 40x). We counted three fields per section and three sections per animal for control and EtOH groups (Fig. 1).

### Biochemical analysis

After behavioural assays, a different set of animals from that used in histological studies (n = 5 animals per group) were sacrificed by cervical dislocation and brains were immediately removed and cooled on dry ice. Then, the entire cerebral cortex was separated from the rest of the encephalon and homogenized in ice-cold 20 mM Tris-HCl buffer (pH 7.4).

#### Nitrite assay

An aliquot of crude homogenate was centrifuged at 21,000 *g* for 20 min at 4°C, and supernatant was used to analyze nitrite levels as described elsewhere [27]. Briefly, samples were incubated at room temperature for 20 min with Griess reagent [0.1% N-(1-naphthyl) ethylenediaminedihydrochloride; 1% sulfanilamide in 5% phosphoric acid; 1∶1]. The absorbance was measured at 550 nm and compared to that of standard solutions of sodium nitrite.

#### Lipid peroxidation (LPO) assay

Lipid peroxidation was evaluated by measuring malondialdehyde (MDA) and 4-hydroxyalkenals (4HDA) levels as previously showed [28]. Briefly, an aliquot of crude homogenate was centrifuged at 2,500 *g* for 30 min at 4°C, and supernatant was processed as described by the Bioxytech LPO-568 kit (Cayman Chemical). This kit takes advantage of a chromogenic reagent that reacts with MDA and 4HDA at 45°C, yielding a stable chromophore with maximal absorbance at the 586 nm wave length.

#### Protein content

Quantities of proteins in the supernatants (used for determination of lipid peroxidation and nitrite levels) were assayed as described elsewhere [29]. Thus, after corrected for protein concentration, results of lipid peroxidation and nitrite levels were expressed as percentages of control groups.

### Statistical analyses

All values are expressed as means±S.E.M. (n = 10 animals per group for behavioral tests and n = 5 per group for immunohistochemistry and biochemical analyses). Statistical comparison of body weight gain between control and EtOH groups was performed using one-way analysis of variance (ANOVA) with repeated measures (days). Data from rotarod test were evaluated with one-way ANOVA (treatment) with repeated measures (trials). Following significant ANOVAs, multiple post-hoc comparisons were performed using the Tukey’s test. The rest of data was compared using Student’s t-test. The accepted level of significance was P<0.05. All tests were performed using the Statistica software package (StatSoft Inc., Tulsa, OK, USA).

## Results

### Effects of chronic EtOH exposure during adolescence on body weight gain in rats

**Figure 2:**
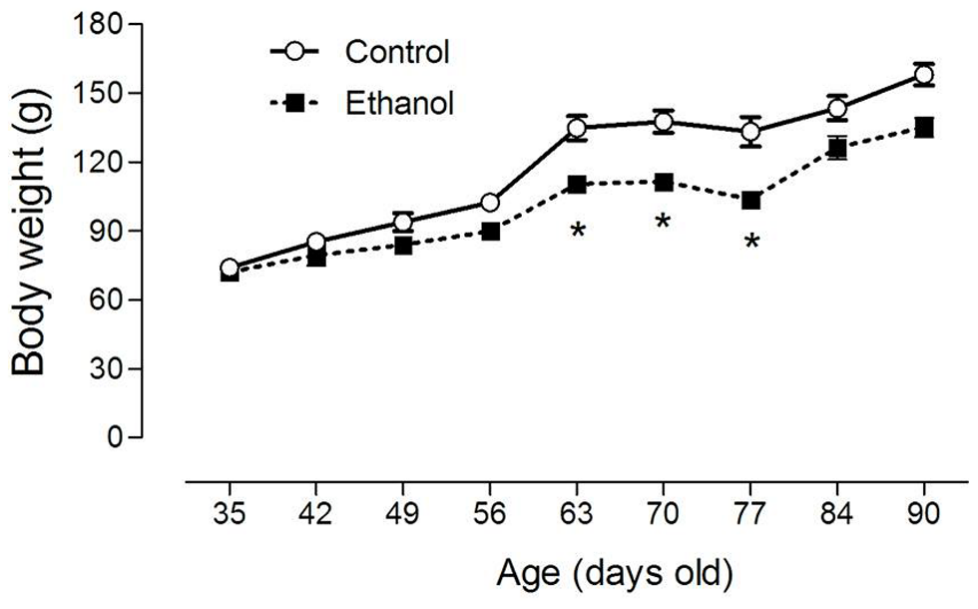
Effects of chronic ethanol (EtOH) administration (6.5 g/kg/day) during adolescence over a period of 55 days (i.e., from the 35th day until the 90th day of life) in the body weight gain of rats. The results are expressed as mean ± SEM of the body weight with interval of 7 days (n = 10 animals per group). *P<0.05 compared to control group (One-way ANOVA with repeated measures followed by Tukey’s test).

As illustrated in Fig. 2, the body weight of the animals was evaluated during the entire period (55 days) of ethanol exposure (i.e., from the 35^th^ day until the 90^th^ day of life) at intervals of 7 days. One-way ANOVA with repeated measures revealed significant effects for the treatment factor (F_1,16_ = 73.11; *P*<0.0001), for the repetition factor (F_8,128_ = 84.67; *P*<0.0001) and for the interaction factor between treatment and repetition (F_8,128_ = 2.68; *P*<0.05). Subsequent post-hoc comparisons using the Tukey’s test revealed that ethanol induced a significant reduction in the body weight gain of rats specifically from the 63^th^ to 77^th^ days of life (*P*<0.05). However, the final body weight of control- and EtOH-treated rats after the period of 55 days of treatment did not differ statistically (Fig. 2).

### Chronic EtOH exposure during adolescence disrupts the motor performance of rats

**Figure 3:**
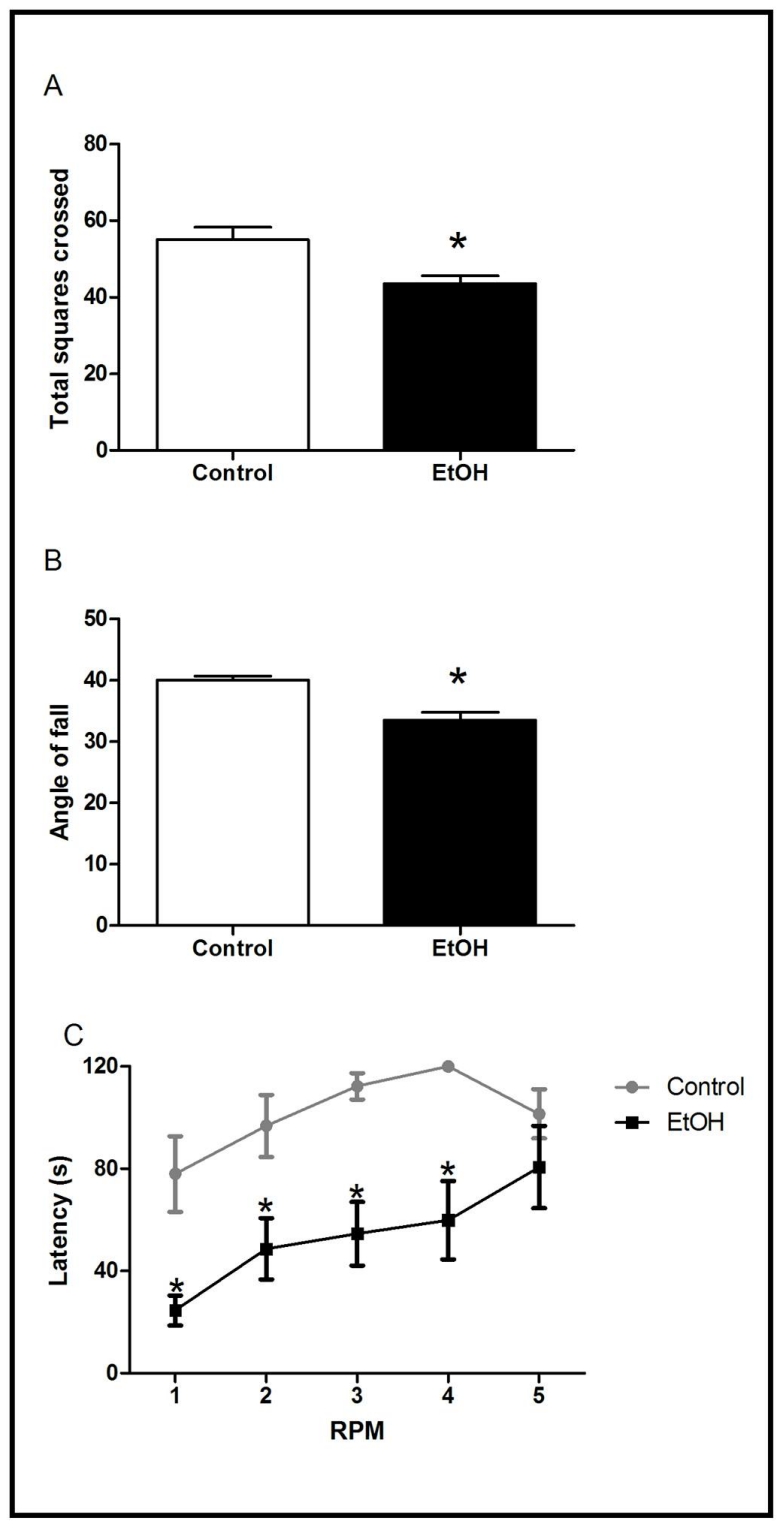
Effects of chronic ethanol (EtOH) administration (6.5 g/kg/day) during adolescence on the locomotor activity of male Wistar rats evaluated in the open field (OF), inclined plane and rotarod test. The results are expressed as mean ± SEM of the: (A) total squares crossed (OF test); (B) Angle of fall (inclined plane test); (C) Latency to fall during 5 sessions at 15 rotations per minute (rotarod test) (n = 10 animals per group). *P<0.05 compared to control group (t-Student test for A and B; One-way ANOVA with repeated measures followed by Tukey’s test for C).

The results of locomotor activity evaluated in the open field arena (for 5 min) are summarized in Fig. 3A. Student’s t-test indicated a significant reduction (P<0.05) in the total number of squares crossed by EtOH-treated rats in the open field test.

The effects of chronic ethanol administration during adolescence on the rats’ ability to maintain postural stability evaluated in the inclined plane test are illustrated in Fig. 3B. Student’s t-test comparisons indicated that EtOH administration induced a significant decrease in the mean angle score when compared to the control group (P<0.05).

The effects of chronic ethanol administration during adolescence on the motor coordination and balance of rats evaluated in the rotarod test are illustrated in Fig. 3C. One-way ANOVA with repeated measures revealed significant effects for the treatment [F_2,54_ = 76.85; *P*<0.0001] and the repetition factor [F_2,108_ = 29.33; *P*<0.0001] and the interaction factor between treatment and repetition [F_4,108_ = 4.59; *P*<0.01] in the latency to fall of the rotarod. Post-hoc comparisons revealed that ethanol exposure during adolescence induced a significant decrease in the latencies to fall in the four test sessions of the rotarod (Fig. 3C).

### Chronic EtOH exposure during adolescence induces neuronal loss and reduction in the number of microglial and astrocyte cells in the cerebral cortex of rats

**Figure 4:**
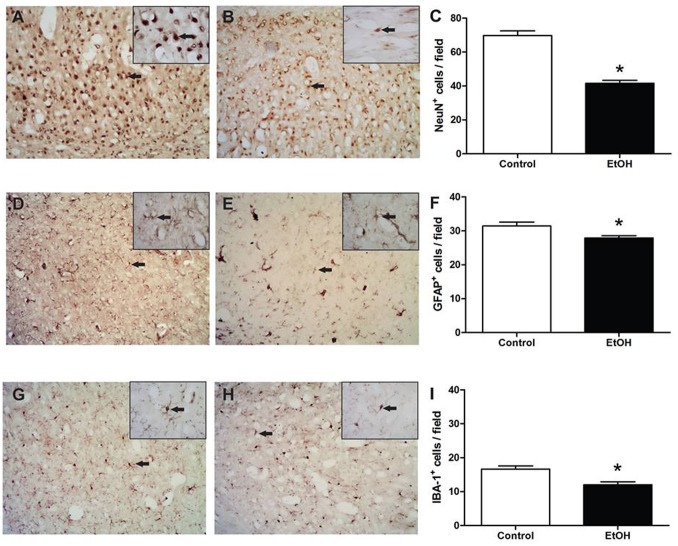
Effects of chronic ethanol (EtOH) administration (6.5 g/kg/day) during adolescence on the cell density of cortex motor. These results are expressed with photomicrographs and mean ± SEM. A (Control), B (EtOH), C: photomicrographs and graph expressing Neu-N label; D (Control), E (EtOH), F: photomicrographs and graph expressing GFAP label; G (Control), H (EtOH), I: photomicrographs and graph expressing Iba-1 label (n = 5 animals per group). *P<0.05 compared to control group (t-Student test). Scale = 200 µm. Inset, scale = 100 µm.

The immunohistochemistry for neuronal cells (NeuN) identified that EtOH induced neuronal loss in the cerebral cortex of rats (Fig. 4 A–C).

After 55 days of ethanol exposure, it was observed a significant reduction in the number of GFAP+ cells revealed an expressive reduction in astrocyte cells in rats treated chronically with EtOH during adolescence (Fig. 4 D–F).

Moreover, a quantitative assessment of microglial measured by Iba-1+ cells labelled in the cerebral cortex revealed an expressive reduction of this cell (Fig. 4 G–I).

### Chronic EtOH exposure during adolescence increases nitrite levels and lipid peroxidation in cerebral cortex of rats

**Figure 5:**
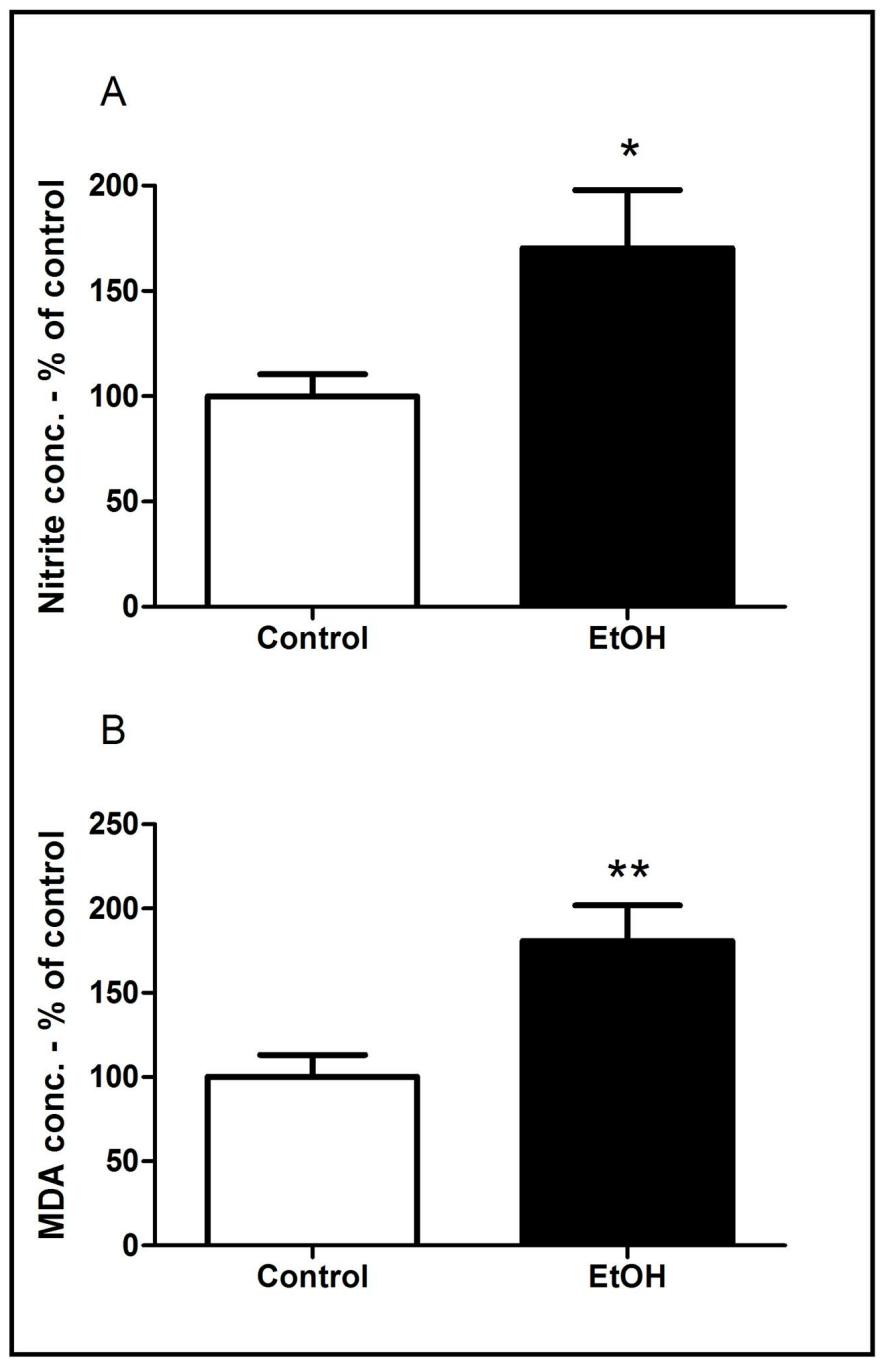
Effects of chronic ethanol (EtOH) administration (6.5 g/kg/day) during adolescence on the oxidative stress parameters of Wistar rats. The results are expressed as mean ± SEM of the: (A) Nitrite concentration per milligram of protein; (B) Malondialdehyde (MDA) concentration per miligrams of protein (n = 5 animals per group). *P<0.05 compared to control group (t-Student test).

Chronic exposure to ethanol during adolescence induced an increase in oxidative stress-related parameters in cerebral cortex of rats, as evidenced by a significant increase in both nitrite levels (an indirect marker of nitric oxide production) (Fig. 5A) and lipid peroxidation (Fig. 5B).

## Discussion

The present study demonstrates, for the first time, that chronic EtOH intoxication during adolescence induces marked long-term motor disruption evaluated in different behavioral tasks (open field, inclined plane and rotarod) that were accompanied by cellular alterations in the cerebral cortex of rats. Additionally, biochemical assays revealed a main role for oxidative stress, and especially for nitrergic pathway in the present effects induced by chronic EtOH exposure during adolescence.

Epidemiological studies have been shown the increasing consume among adolescents in the world, that initiates around the 12 years old and increasing during overall adolescence, reaching about 45.5 % in young adults [30]. A recent survey about EtOH consume in Brazil has indicated similar data from that observed in other countries in which the consumption of alcohol is increasing among adolescents and in a heavy drinking profile [2].

Early evidence revealed that EtOH induces brain damage observed by brain atrophy that was more evident among alcoholics that have drunk heavily and during long-term periods [3]–[5]. Other studies also revealed marked impairments on motor and cognitive functions in both humans and laboratory animals following chronic ethanol exposure (for review see [31]). Indeed, it is well documented that adolescence is a period of vulnerability for drug abuse [32]. However, the long-term neurobehavioral consequences and molecular mechanisms remain poorly understood. It is well documented that the relationship between excessive alcohol consumption and neurotoxic levels among adolescents in a binge drinking episodes highlights to the fact that they are resistant to sedative effects and motor deficits induced by alcohol intake. On the other hand, adolescents have a profile of higher vulnerability to rewarding properties of alcohol when compared to adults [33].

Bell et al. [34] have investigated the acquisition of EtOH drinking behavior during adolescence in selectively bred alcohol-preferring rat model. The authors demonstrated that under 24-h of free-choice paradigm, adolescent alcohol-preferring rats intake 7.5 g/kg of alcohol per day till 10 mg/kg/day when offered multiple concentration of EtOH solution that can reflect the voluntary heavy consume during adolescence. Thus, in the present study we investigated whether heavy chronic ethanol exposure during adolescence induces long-term motor impairments and histological damage in cerebral cortex of rats.

Previous studies have shown that in the adolescence occurs structural and functional maturation of the brain. In this period, there are significant modifications in brain organization and cortical circuitry [35]. Alcohol inhibits brain maturation and neurogenesis, becoming adolescent brain more susceptible to the neurotoxic effects of alcohol abuse [36]. Motor coordination impairments following acute EtOH doses are more prominent in adult than adolescent rats. However, chronic alcohol exposure is more harmful during adolescence, mainly on motor function, that persists long-term after stopping alcohol administration [37], [38].

The results of the present study showed that chronic EtOH exposure during adolescence affects locomotor activity of rats, as indicated by reduction in the number of squares crossed in the open field arena. Pascual et al. [39] demonstrated that adolescent rats exposed to EtOH (3.0 g/kg/day) for two consecutive days at 48-h intervals over 14 days display motor deficits related to cerebellar and neocortex damage by inflammatory mechanisms. We also observed that rats chronically exposure to EtOH during adolescence displayed a disruption in their ability to maintain postural stability and motor coordination on rotarod. These findings are in accordance with a recent study of our group [14] showing that chronic EtOH administration (6.5 g/kg/day during 55 days) during adolescence may potentiate the motor impairments and motor cortex damage induced by focal ischemia in female rats.

The motor cortex has long been viewed to play an important role in fine motor control and fractionation of movement [40], [41], sensorimotor integration and higher-order cognitive-motor movements [42]. There are also other studies showing the role of motor cortex in the performance of behavioral tasks utilized in the current study [43] as well as showing that alterations on motor cortex can be associated with impaired spontaneous locomotion and incoordination in rodents following ethanol exposure [14], [44].

Previous immunohistochemical studies demonstrated that alcohol exposure was related to neuronal loss in cortical regions [14], [16], [45], which is in agreement with our data showing reduction in the anti-NeuN+ cells in the cerebral cortex. The mechanisms associated to ethanol-induced neuronal death in previous studies have been inferred as consequence of increase of oxidative stress and induction of pro-inflammatory mediators including cytokines, COX-2 and iNOS [12], [14], [16], [23], [24].

On the pathophysiology of ethanol intoxication, microglial activation seems to represent a pivotal step by releasing pro-inflammatory factors [22], [46]. Moreover, during ethanol intoxication, there is an increase of nuclear factor kappa B (NF-kB) transcription [47] that plays a role in the signaling of immune and inflammatory responses [48], and a reduction of protein dependent of adenosine monophosphate cyclic (AMPc) family transcription [47] with neuroprotective functions against oxidative stress, excitotoxicity and apoptose [47], [49], [50].

Previous experimental [36], [51] and *post-mortem* brain human studies [20] have shown that alcohol increases the expression of microglial markers after chronic alcohol exposure. Interestingly, and in accordance with previous findings from literature [10], we observed that chronic ethanol exposure during adolescence reduced microglial density in cerebral cortex of rats. Therefore, the current data suggests that ethanol exposure appears interfered with microglial proliferation.

It must be considered that microglial activation may also be related to neuroprotective events following stroke [52] and ethanol intoxication [53]. For instance, 3D two-photon microscopy analysis revealed that following oxygen-glucose deprivation (OGD) in hippocampal slices, microglia physically interacts with neurons protecting these cells from ischemic damage [54]. The same group showed that microglia provides protection following ischemia by phagocytosing [55]. In addition, genetic ablation of proliferating microglia exacerbates the ischemic damage, manifested by increases pro-inflammatory cytokines, apoptosis and infarct areas following middle cerebral artery occlusion (MCAO) in mice [52].

Following ethanol intoxication, microglia may serve both neuroprotective and detrimental actions [53]. While in the acute phase of ethanol intoxication microglia were shown to contribute to damage, they seem to be neuroprotective in the withdrawal phase [53]. Consequently, anti-inflammatory approaches must be devised to maximize the beneficial actions of neuroinflammation, while reducing their detrimental effects.

Moreover, our study demonstrated that alcohol reduced astrocyte population that was contradictory to other studies that inferred that alcohol increased astrocytes cells linked to increase production of reactive oxygen species (ROS) and calcium releasing in mitochondria of these cells [18], [19], [56]. In this sense, astrocytes protect against oxidative stress induced by alcohol perhaps by antioxidant factors production, such as glutathione peroxidase [57].

In addition to neuroinflammatory effects in binge alcohol-induced neuronal degeneration, oxidative stress has been largely associated to ethanol intoxication [58]. In cerebral cortex, chronic intake of alcohol alters cell membrane fluidity probably due to oxidative stress as indicated by increased nitrite and lipid peroxidation levels, enhanced presence of nitric oxide synthase and decreased superoxide dismutase [59]. However, all these effects were described for adult brains, and deleterious consequences for the developing brain with chronic intoxication from adolescence to adult age remains unknown.

Recently, damage by oxidative stress was showed in adolescent mice chronically exposed to alcohol for two weeks [60]. Interestingly, although this exposure caused a significant protein oxidative damage to hippocampus, no alterations were detected in prefrontal cortex [60]. By opposite, our data indicates that longer periods of exposure (55 days, from adolescence to adult age) lead to significantly raise both nitrite and lipid peroxidation levels in cerebral cortex of rats. These results are according to those described elsewhere in hippocampus and cerebral cortex of adult animals intoxicated with ethanol [61]. Therefore, we hypothesized that in adolescent rodents, a marked oxidative stress can be triggered initially in hippocampus, while longer periods of ethanol exposure are required to detect oxidative stress parameters in cerebral cortex. This constitutes a very interesting field that requires additional research.

## Conclusions

Our results provide new evidence that heavy chronic ethanol intake during adolescence in male rats promotes long-term motor coordination deficits, as well as decreased spontaneous locomotion and balance. These behavioral changes were accompanied by a significant reduction in number of neurons and glia cells with increase in both nitrite levels (an indirect marker of nitric oxide production) and lipid peroxidation in the cerebral cortex. The exact mechanisms involved in reduction of glia cells remain unclear and more work is needed to understand how decrease in glia occurs in response to ethanol during adolescence.
